# The Innovation Comes from the Sea: Chitosan and Alginate Hybrid Gels and Films as Sustainable Materials for Wastewater Remediation

**DOI:** 10.3390/ijms21020550

**Published:** 2020-01-15

**Authors:** Maria Laura Tummino, Giuliana Magnacca, Dafne Cimino, Enzo Laurenti, Roberto Nisticò

**Affiliations:** 1 Department of Chemistry, Università degli studi di Torino, Via P. Giuria 7, 10125 Torino, Italy; marialaura.tummino@unito.it (M.L.T.); giuliana.magnacca@unito.it (G.M.); dafne.cimino@unive.it (D.C.); enzo.laurenti@unito.it (E.L.); 2 NIS Centre, Università degli studi di Torino, Via P. Giuria 7, 10125 Torino, Italy; 3 Department of Environmental Sciences, Informatics and Statistics, Università Ca’ Foscari di Venezia, Via Torino 155, 30172 Venezia-Mestre, Italy; 4 Department of Applied Science and Technology DISAT, Polytechnic of Torino, C.so Duca degli Abruzzi 24, 10129 Torino, Italy

**Keywords:** alginate, biopolymers, biowaste valorisation, chitosan, hydrogels, wastewater remediation

## Abstract

The growing utilization of renewable and residual biomasses for environmental preservation and remediation are important goals to be pursued to minimize the environmental impact of human activities. In this paper, sodium alginate (derived from brown algae) was crosslinked using chitosan (mainly derived from the exoskeleton of crustaceans) in the presence of biowaste-derived substances isolated from green compost (BBS-GC), to produce hydrogels and dried films. The obtained materials were tested as adsorbents for wastewater remediation. To this purpose, gels were characterized using a multi-analytical approach and used as active substrates for the removal of three differently-charged molecules, chosen as model pollutants: crystal violet, rhodamine B, and orange II. The effectiveness of the gel formulations was demonstrated and attributed to the variety of active functionalities introduced by the different precursors, the structural factors and the peculiar physicochemical properties of the resulting materials.

## 1. Introduction

In the last few years, the need for environmental preservation is becoming urgent, since pollution and the connected climate change threaten entire ecosystems. This emergency has implications for people’s health and safety, as well as for social equality and the economy. The scientific community, together with entities such as the United Nations [1], are working to solve these concerning issues. Among the main goals, it is possible to mention the sustainable management of water and sanitation, as well as the reduction, reuse and recycling of waste and the use of renewable sources for the production of energy and materials [1,2]. 

In this work, renewable and waste-recovered substances were employed to prepare hydrogels and films (by hydrogel drying) to be applied as pollutant adsorbents. Alginate and chitosan, polysaccharides derived from the marine environment, and biowaste-derived substances were involved in the preparation of such materials.

**Scheme 1:**
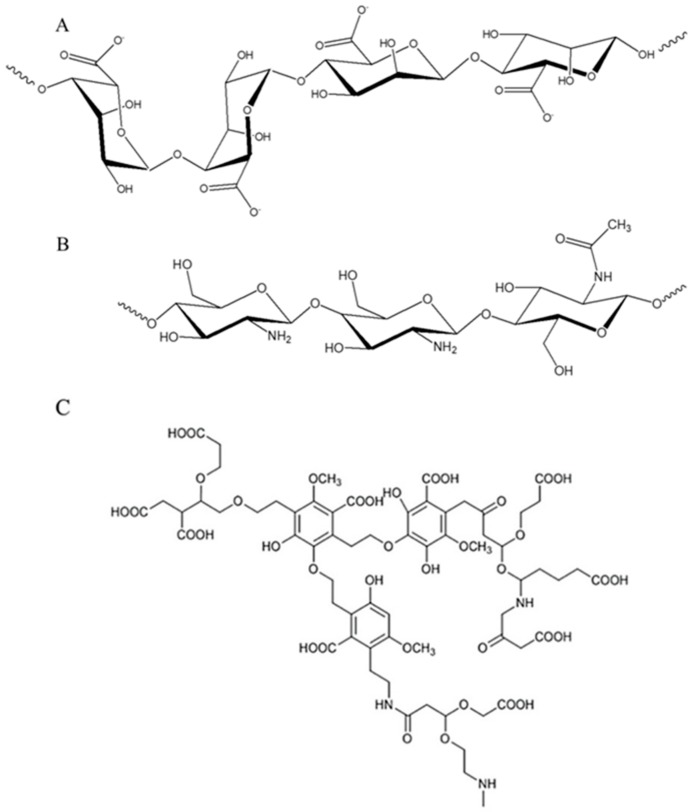
Chemical structures of (**A**) alginate, (**B**) chitosan and (**C**) model of composted bio-waste substances.

Alginates are linear copolymers made by (1–4)-linked residues of β-d-mannuronic acid and α-l-guluronic acid units (Scheme 1A), isolated from the cell walls of various species of brown algae (*Phaeophyceae*) [3,4], which are traditionally harvested and used also in industrial productions [5,6]. In alginate structure, abundant free hydroxyl and carboxyl groups are distributed along the backbone chain of the polymer [7]. Since alginates are biocompatible, biodegradable, non-toxic, stable in the environment and have strong gelation, film-forming, and complexing abilities [7], they have been used as biomaterials for several applications including tissue engineering scaffolds, drug delivery systems, wound dressings, antibacterial agents [3], immobilized biocatalysts (carrying enzymes), as multifunctional additives within food and beverage industries [8], and for energetic [9] and environmental remediation purposes [7,10].

Chitosan is a random copolymer made using β-(1→4)-linked 2-acetamido-2-deoxy-d-glucopyranose and 2-amino-2-deoxy-d-glucopyranose units (Scheme 1B) and is obtained by alkaline *N*-deacetylation of chitin, which is the main component of the exoskeleton of crustaceans, such as shrimps, crabs and lobsters [11,12,13]. Considering that a major percentage of processed seafood consists of crustaceans and the wastes produced by their exoskeleton and cephalothoraxes represent 50%–70% of the weight of the raw material, the extraction of components contained in these scraps is a convenient way to produce valuable resources, applicable in different fields [13,14]. Chitosan has unique properties among biopolymers since it contains primary amino groups [14] and is considered a low-toxic, non-immunogenic and biodegradable polysaccharide [15]. Indeed, chitosan and its derivatives have been used in the cosmetic industry, for food packaging, agriculture, environmental remediation and biomedical applications such as drug-delivery systems and/or as antimicrobial agents [16,17,18,19].

Alginate and chitosan are very promising precursors capable of forming gels under very mild conditions, namely at room temperature (RT) and in the absence of toxic solvents [11,20], i.e., through a simple ionic crosslinking or complexation [21,22]. In general, hydrogels are made by hydrophilic macromolecular chains organized in a polymeric three-dimensional network (or *gel*) with visco-elastic properties, obtained directly from a colloidal aqueous solution (or *sol*) at low temperature [21]. In order to obtain a stable three-dimensional structure (thus avoiding a partial/complete dissolution of the 3D network or its collapse), the presence of crosslinks between the polymeric chains/segments forming the hydrogels is mandatory [21]. Depending on the kind of crosslinks, hydrogels are classified as physical or chemical gels. Physical gels are networks held together by molecular entanglements and/or secondary forces including ionic and H-bonds or hydrophobic interactions. Conversely, in chemically crosslinked gels, covalent bonds are present between the different polymer chains forming the network (linked by addition or condensation reactions, by chemical reactions with aldehydes, or through high energy irradiation) [15,21,23]. In particular, gels made by mixing alginate and chitosan have been carefully studied as results of the cooperative electrostatic interaction between poly-cationic chains of chitosan and poly-anionic chains of alginate [24]. Furthermore, hydrogels can be easily converted into their dried forms (xerogels) by the evaporation of the swelling agent (water) [25,26]. This way, they can enhance their absorption capacity raising their dry weight up to three orders of magnitude, when re-soaked in the aqueous medium.

Concerning this work, it is worth specifying that both alginate and chitosan-based gels have been already widely exploited as adsorbents in water remediation, due to the presence of carboxylic acids and amine groups [27], which can act as chelation/coordination sites for the removal of heavy metals, dyes and emerging pollutants [10,28]. 

Despite the wide number of studies performed on alginate and chitosan hydrogels, the preparation of new materials with improved features and performances is a cutting-edge theme. Herein, the additive chosen and tested for enhancing the adsorption capacity of the hydrogels is named BBS-GC, with the acronym corresponding to bio-based substances isolated from green composted biowaste [29]. It is made of water soluble macromolecules with a complex lignin-derived structure containing acid (i.e., carboxylic groups) and basic functional groups (i.e., amino group) bonded to aromatic and aliphatic chains, plus a portion of ashes (a similar structure found in a previous work [30] is reported in Scheme 1C) [29,31]. The large amount of freely available functionalities makes BBS-GC an interesting adsorption promoter, as already demonstrated in previous studies on other systems [32,33].

Although many types of substances represent concerning contaminating agents for water (such as pharmaceuticals, pesticides, heavy metals, etc…) the scientific community is currently investigating the phenomenon of dye pollution. This fact can be demonstrated by the huge amount of studies published in the last five years (about 13,000 results on Scopus database including “*dye pollution*” and “*dyes adsorption*” as keywords). The issue can be mainly correlated to textile manufacturing, which is in constant evolution and activity, as recently reported in some reviews [34,35,36]. The problems caused by dyes fall in two main fields: environment (i.e., affecting the photosynthetic function in plants and eutrophication of aquatic life [37]) and human health [38]. 

Therefore, in this study hydrogels/dried films were prepared by ionically crosslinking sodium alginate and chitosan alone or englobing BBS-GC to improve the final adsorbing features towards three dyes as model pollutants: orange II (negatively-charged), rhodamine B (in zwitterionic form) and crystal violet (positively-charged). The azo dye orange II is widely used in a variety of industries ranging from textile to paper, and its toxicity is comparable to other highly toxic compounds (i.e., phenols and metals) [39]. Rhodamine B represents another well-known dye used in the textile industry due to its high stability, and its release into the environment is dangerous for aquatic life [40]. In California, rhodamine B is suspected to be carcinogenic, so products containing it must present a warning on the label [41]. Lastly, crystal violet is extensively used to dye different kinds of textiles and paper [42], to produce printing inks and represents a dermatological agent in veterinary medicine [43]. It is toxic, may be absorbed through the skin causing irritation and is harmful by inhalation and ingestion [38,43,44]. In extreme cases, it can lead to kidney failure, eye irritation until permanent blindness and cancer [38,43,44]. 

For the sake of completeness, the prepared formulations were coded as H (hydrogels) or F (dried films), followed by “Alg”, “Chito” and “BBS” as abbreviations for alginate, chitosan and BBS-GC.

## 2. Results and Discussion

### 2.1. Physicochemical Characterizations

**Figure 1:**
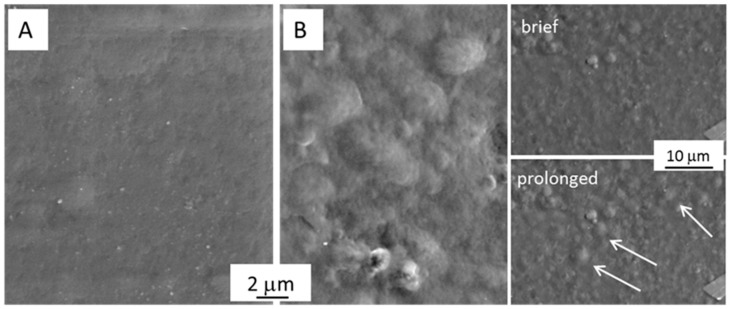
SEM micrographs showing the surface of (**A**) F-Alg/Chito and (**B**) F-Alg/Chito/BBS. The inset in section B reports the aspect of the surface after brief (few seconds) and prolonged (ca. 1 min) exposure to the electron beam (the arrows evidence the defects formed during the exposure).

Gels were prepared by mixing alginate with chitosan (with or without BBS-GC): BBS-GC-containing gels were brown-coloured, whereas the reference samples were almost colourless. These samples were washed several times with ultrapure water to remove unreacted substances. Moreover, since chitosan requires a weak acid environment to be solubilised, correction of the pH from acid to neutral was mandatory for all Alg/Chito-based gels, to avoid any effect of the acidity compromising the adsorption capacity of the final materials. The neutralization for Alg/Chito-based gels occurred after six rinsing cycles, for a global duration of 60 min, with a simultaneous BBS-GC loss of ca. 10 wt.% (also visible in the yellowish coloration of the water after every rinsing cycle). This neutralization step was monitored following pH variation after every washing step until reaching a final pH value of 7.0 ± 0.5. The morphology of dried films was evaluated using scanning electron microscopy (SEM) analysis. In general, the surface of F-Alg/Chito appeared compact and almost smooth (Figure 1A) in accordance to the literature [45] for a xero-polyelectrolyte complex constituted by alginate and chitosan. In the case of F-Alg/Chito/BBS (Figure 1B), several irregularities appeared, which increased by sample exposure to the electron beam, as indicated by the white arrows in the inset of Figure 1B, where the same region after brief and prolonged exposure to the beam is shown. The peculiar molecular organization caused by the complex interaction of BBS-GC in the 3D alginate-chitosan network seems to have generated a more disordered structure and consequently a more limited stability. XRD analyses performed on F-Alg/Chito and F-Alg/Chito/BBS revealed an amorphous structure (data not reported for the sake of brevity).

**Figure 2:**
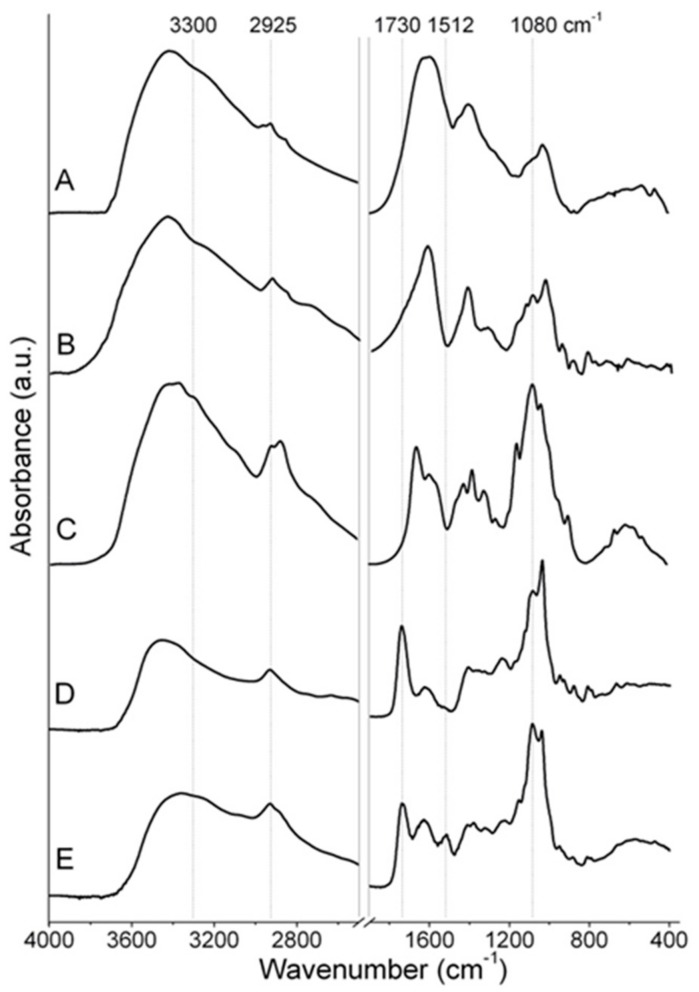
FTIR spectra of (**A**) BBS-GC, (**B**) sodium alginate, (**C**) chitosan, (**D**) F-Alg/Chito and (**E**) F-Alg/Chito/BBS. Dotted lines evidence the position of some significant signals described in the main text.

Fourier transform infrared (FTIR) spectroscopy was used to evaluate the vibrational features of the dried films (Figure 2). Due to the similar absorption ranges of the three bio-substances (i.e., alginate, chitosan and BBS-GC), it was difficult to clearly assign the IR bands of the films to each specific precursor. The BBS-GC IR spectrum (Figure 2, curve A) presented signals at ca. 3400 cm^−1^ ascribable to O-H stretching mode and a weak signal at 2925 cm^−1^ due to C-H stretching mode; carboxylic and amide groups (symmetric stretching vibrations of C=O) were present at 1630 cm^−1^, whereas signals at 1420 cm^−1^ were correlated to C-H bending and/or O-C-O stretching of carboxylate groups; signals at 1034 cm^−1^ were due to C-O-C stretching and/or aryl-bonded C-H bending [32]. 

The main IR signals in the spectrum of neat sodium alginate (Figure 2, curve B) were: a strong and broad band due to the axial O-H stretching mode centred at ca. 3400 cm^−1^ attributable to the presence of either O-H groups or water molecules interacting via H-bonding, a complex signal at 2920–2850 cm^−1^ due to C-H stretching modes, the absorption bands centred at 1630 cm^−1^ and 1460 cm^−1^ attributable to asymmetric and symmetric stretching vibrations of O-C-O groups [46], respectively, and the bands in the 1107–940 cm^−1^ range due to C-O and C-O-C stretching modes of pyranosyl rings [47,48]. 

Analogous signals were present in the high-frequency region for neat chitosan (O-H and C-H stretching modes, Figure 2, curve C) accompanied by some weak signals at ca. 3300 cm^−1^ due to N-H stretching vibrations. In the spectrum of chitosan it was possible to observe as well: the band centred at 1655 cm^−1^ attributable to the C=O symmetric stretching mode (amide I), the one at 1580 cm^−1^ due to the angular deformation of N-H bonds of the amino groups [49], the peaks in the 1420–1477 cm^−1^ range resulting from the coupling of the C-N axial stretching and N-H angular deformation [12] and the sharp band at 1377 cm^−1^ ascribable to the CH_3_ symmetrical deformation mode [49,50]. Finally, chitosan fingerprint bands due to skeletal signals in the 1150–900 cm^−1^ range were attributable to C-O and C-O-C stretching vibrations [51].

The comparison between F-Alg/Chito and F-Alg/Chito/BBS (D and E curves, respectively in Figure 2) showed the main differences between the two samples principally positioned in the region of carboxylate and amide signals between 1500 and 1750 cm^−1^. The observed bands are due to the interaction of these groups in the physical gels, formed through cooperative interaction between poly-cationic chains of chitosan with the poly-anionic chains of alginate. As already observed in the literature, the ionic interactions between the COO^-^ and NH_3_^+^ groups can be ascertained by both the formation of signals at 1080 cm^−1^ [48] and at ca. 1730 cm^−1^ [46,52]. In particular, the latter was assigned to the carboxylate stretching in the new poly-ion complex [46,52]. Additionally to this evidence, a drastic reduction of the signal at ca. 3300 cm^−1^ was observed with a change in the shape (less intense, larger and not well defined), which possibly reflects the formation of physical interaction randomly established between the chitosan amino groups and the alginate carboxylate ones. Lastly, the small peak at 1512 cm^−1^ in the spectrum of F-Alg/Chito/BBS (Figure 2E) seems to be mostly related to the presence of BBS-GC, which showed an intense and broad absorbance in that spectral region, although the very complex structure of BBS-GC makes the assignation of this signal to a specific interaction very difficult.

**Figure 3:**
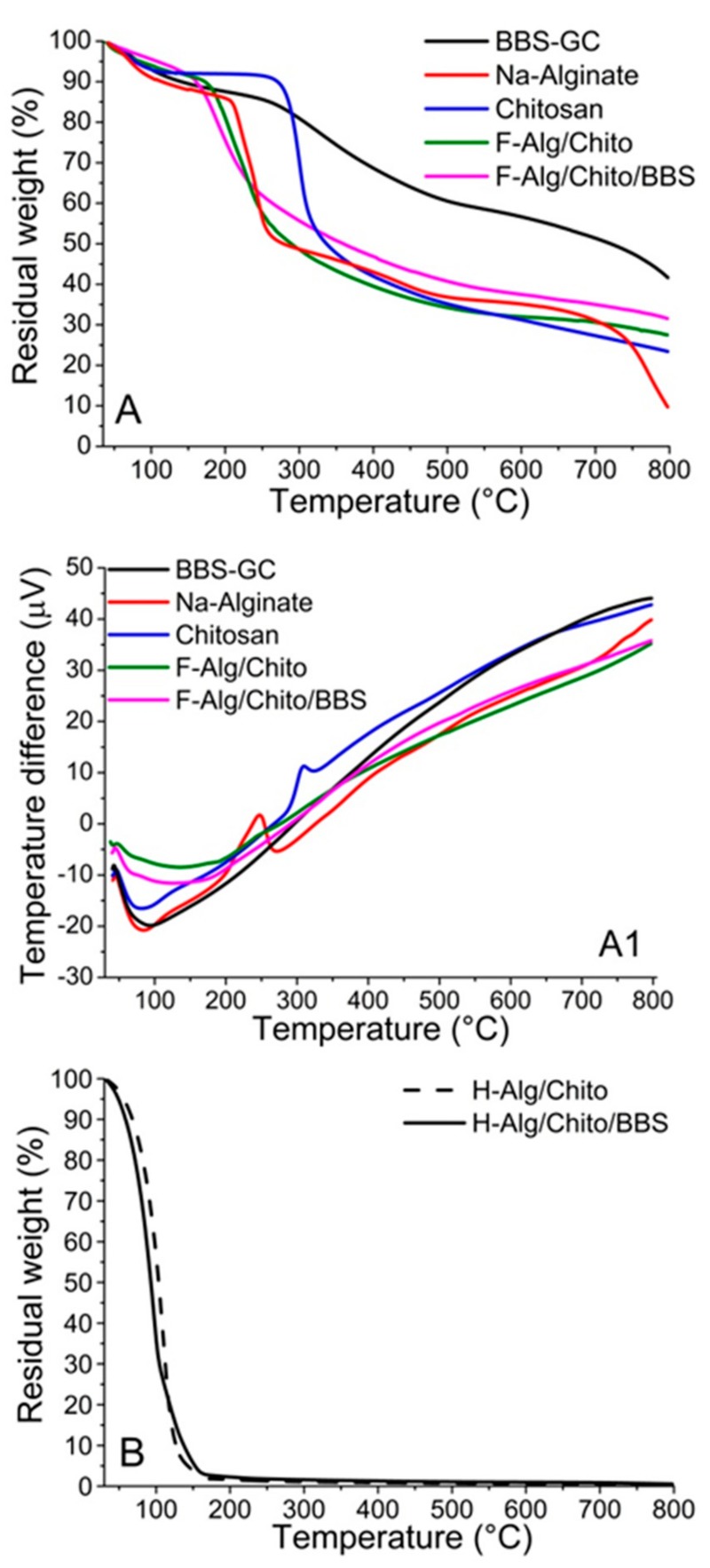
(**A**) Thermal gravimetrical analysis (TGA) and (**A1**) Differential thermal analysis (DTA) profiles of BBS-GC (black curve), sodium alginate (red curve), chitosan (blue curve), F-Alg/Chito (green curve) and F-Alg/Chito/BBS (magenta curve); (**B**) TGA profiles for hydrogels: H-Alg/Chito/BBS (solid curve) and H-Alg/Chito (dashed curve).

The thermal stability of reference materials (sodium alginate, chitosan and BBS-GC) and related dried gels was studied using thermal gravimetrical analysis (TGA) under nitrogen atmosphere (Figure 3A). The thermal degradation of BBS-GC (Figure 3A, black curve) showed three steps: the first one due to water evaporation at ca. 100 °C and the second one mainly occurring in the range 250–600 °C, but continuing up to 800 °C, involving the decomposition and carbonization of the organic matrix. As expected, the BBS-GC curve showed an important residue (*ca.* 41 wt.%) at 800 °C due to inorganic ashes and carbon from pyrolysis. Concerning sodium alginate (Figure 3A, red curve), after dehydration at around 100 °C, degradation advanced with the formation of Na_2_CO_3_ and carbonization at temperatures higher than 200 °C [53]. The final step at T>700 °C corresponded to the earliest decomposition of Na_2_CO_3_ [54], which still left an inorganic residue of approximately 11 wt.% at 800 °C. The thermal profile of pure chitosan (Figure 3A, blue curve) demonstrated a first weight loss between 50–100 °C due to physisorbed water loss, a second weight loss at around 250 °C due to chitosan degradation and carbonization, occurring with release of CO_2_, CO, NH_3_ and other organic species, and a third slower weight loss at temperatures higher than 450 °C, attributable to the release of CH_4_ due to further dehydrogenation mechanism [12]. In this case, the residue left of approximately 23 wt.% was mainly made of carbon. Lastly, both F-Alg/Chito (Figure 3A, green curve) and F-Alg/Chito/BBS (Figure 3A, magenta curve) displayed a pronounced dehydration step at T<200 °C, followed by a mild degradation profile, with residues of 27 wt.% for F-Alg/Chito and 32 wt.% for F-Alg/Chito/BBS at 800 °C. The gap of 5% between the residues of F-Alg/Chito and F-Alg/Chito/BBS was reasonably ascribed to BBS-GC. In this way, it was possible to estimate the actual BBS-GC amount inside films and, consequently, confirm its slight loss during the cleaning procedure (<10%). Concurrently with TGA, the differential thermal analysis (DTA) profiles, giving information on endo/exothermic transformations involved, are displayed in Figure 3A1. All the samples showed an endothermic peak corresponding to dehydration, in accordance to the weight loss steps observed in TGA graphs: for the three reference materials the phenomenon was completed between 50 and 100 °C, whereas for F-Alg/Chito and F-Alg/Chito/BBS the peak broadened up to 200 °C. In pure chitosan DTA (Figure 3A1, blue curve), an exothermic event is centred at 309 °C, attributable to the degradation and carbonization which started from ca. 250 °C, as detected using TGA. In the case of Na-alginate (Figure 3A1, red curve), two exothermic phenomena can be highlighted: a peak centred at 247 °C, attributable to the progressive transformation of the biopolymer to Na_2_CO_3_, and the starting point of the further decomposition of Na_2_CO_3_, visible at ca. 700 °C.

**Figure 4:**
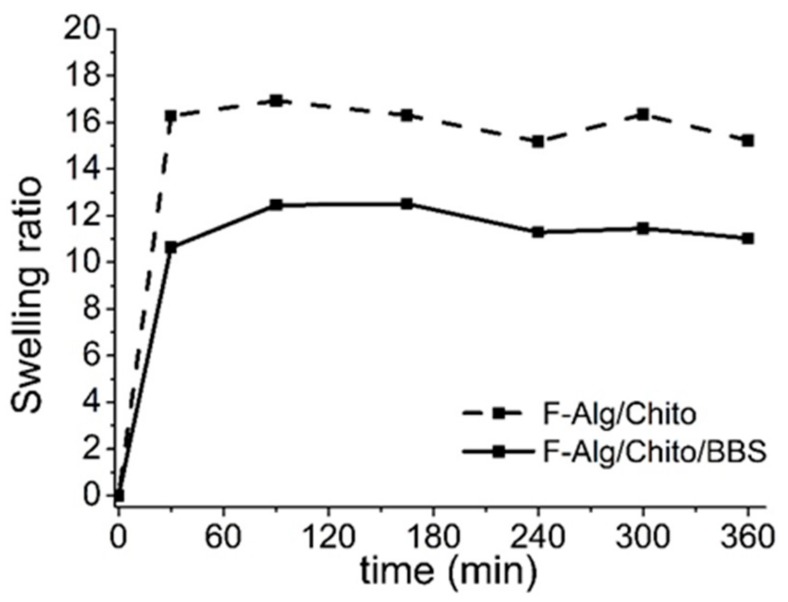
Time dependence of swelling ratios for dried films: F-Alg/Chito/BBS (solid line) and F-Alg/Chito (dashed line).

The thermogravimetric measurements were also used to obtain the amount of water entrapped in the three-dimensional hydrogels (Figure 3B and Table 1), which resulted almost comparable between samples. Regarding the water uptake determined on water-saturated dried films (Table 1) and the swelling ratios studied over 6 h (Figure 4), they are much higher than those found in literature for alginate gels crosslinked with divalent ions (greater than tenfold) [55,56]. In Figure 4, it is possible to observe that equilibrium of the water uptake was reached in about 30 min of contact, and the proportion between the values of the two examined samples remained almost constant throughout the whole experiment. The BBS-GC-containing gels showed lower affinity to water, leading to the hypothesis that the hygroscopic character of chitosan [12] favoured the water retention in the hydrogel, whereas the hydrophobic/amphiphilic moieties of BBS-GC counteracted the chitosan behaviour.

**Figure 5:**
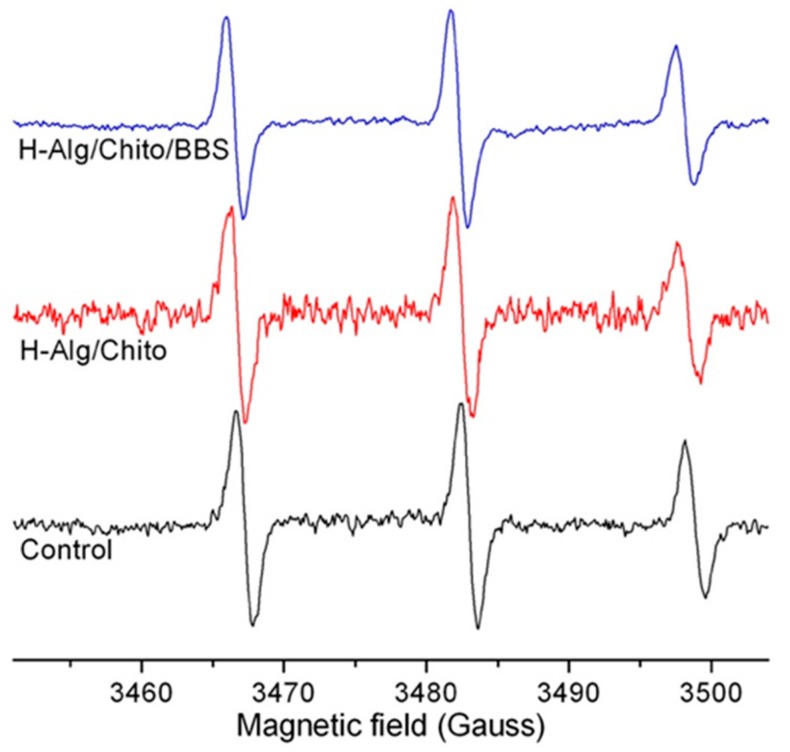
Electron paramagnetic resonance EPR spectra of 5-doxylstearic acid at RT in different environment (intensities were normalized to the central line in order to better compare the spectra).

Electron paramagnetic resonance (EPR) measurements carried out with the spin-label technique were used to assess the structure of the gels looking at the mobility of the spin-label in the forming structure. The obtained results permitted a slight modification of the shape of the nitroxide radical species to be revealed when included in the hydrogels, as shown in Figure 5. Indeed, decrease of spin-label mobility typically induces a widening of the linewidth and a decrease in the height of each spectral line, particularly evident in the high-field line. To distinguish the effects on spin-label mobility, the correlation time (τ_c_) can be calculated by means of simulation software for isotropic and anisotropic systems [57]. Simulation tests showed a very low τ_c_ value for the highly mobile control spectrum (0.086 ns), whereas this value increased for both hydrogels, confirming a reduced mobility for the spin-label in these conditions. In particular, the τ_c_ was higher in the case of the H-Alg/Chito sample (0.123 ns) indicating a more ordered and compact organization of the network with respect to the H-Alg/Chito/BBS gel (0.104 ns). This result could also explain the higher adsorption capacity of BBS-containing gels described in the following paragraph (vide infra) as the more open network could host a higher amount of adsorbates.

**Figure 6:**
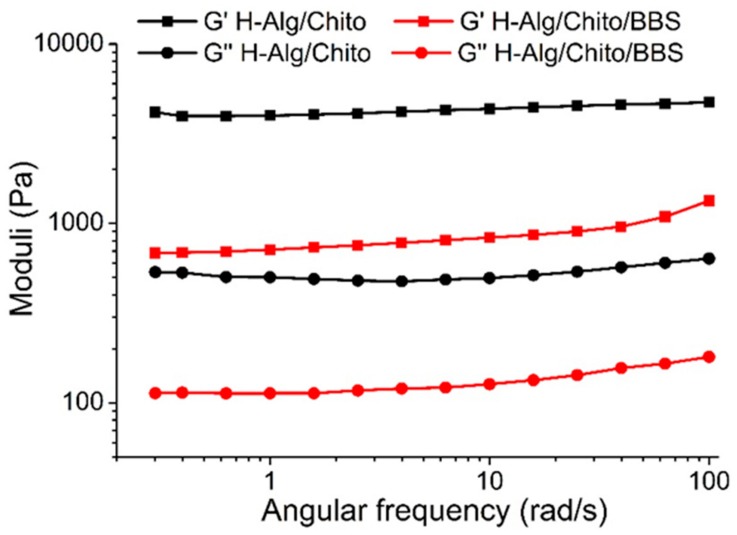
Rheological measurements with indications of the storage G’ (squares) and loss G” (circles) moduli for H-Alg/Chito (black line) and H-Alg/Chito/BBS (red line).

Further investigations concerning the structure of examined hydrogels were carried out using preliminary rheology measurements, as reported in Figure 6. In the frequency range selected for both H-Alg/Chito and H-Alg/Chito/BBS, the storage (elastic) modulus G’ was always higher than the loss (viscous) one G”, meaning that both formulations behave as solid-like gels [58]. The G’ and G” moduli for the same sample remained almost parallel for the whole range of angular frequencies investigated, and the absence of a crossover point indicated that gelation was completed in both cases [59]. H-Alg/Chito demonstrated a greater viscous and elastic behaviour than H-Alg/Chito/BBS, suggesting that the addition of BBS-GC influenced the rheological and, consequently, the mechanical properties of the gels. This evidence can be explained considering that a critical point for the gels’ formation was the viscosity of both Na-alginate and chitosan solutions, whose chains had to slide on each other to physically interact and form the 3D network. In this situation, the complex macromolecules constituting BBS-GC were inserted, changing some interactions among polymeric chains of polysaccharides. In particular, correlating these outcomes with those obtained using EPR, it is possible to hypothesize that in H-Alg/Chito/BBS the molecules of BBS-GC enlarged some points of the net, which is reflected in the higher mobility of the spin-label with respect to H-Alg/Chito.

### 2.2. Adsorption Experiments

**Figure 7:**
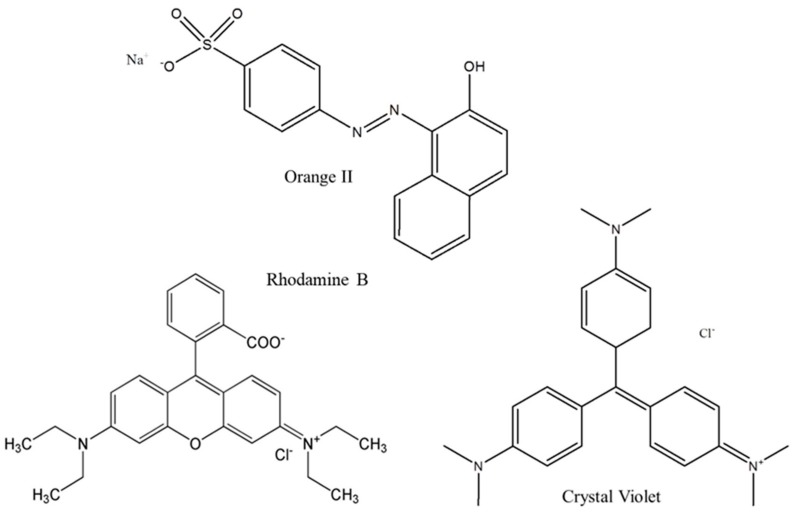
Orange II, rhodamine B and crystal violet chemical structures.

Adsorption behaviour of both hydrogels and dried films were evaluated on different model pollutants for 1 h. Three dyes were selected as model substrates: crystal violet (CV, positively-charged), rhodamine B (RH, in zwitterionic form) and orange II (OII, negatively-charged) (Figure 7). It is worth underlining that the zwitterionic form of rhodamine B in solution is at equilibrium with the cationic one, as already reported by McHedlov-Petrosyan and Kholin [60].

**Figure 8:**
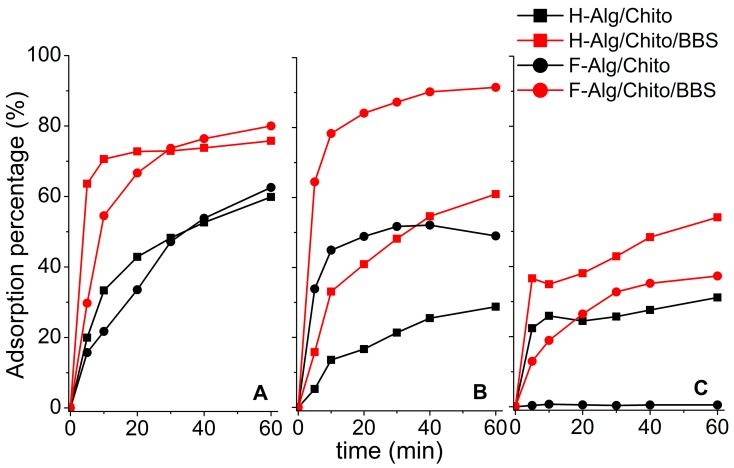
Adsorption percentages of both hydrogels and dried films towards different substrates: (**A**) crystal violet - CV, (**B**) rhodamine B - RH, and (**C**) orange II – OII at initial dye concentration of 10 mg L^−1^. Legend: H-Alg/Chito (black squares, black line), H-Alg/Ca/BBS (red squares, red line), F-Alg/Chito (black circles, black line), and F-Alg/Chito/BBS (red circles, red line). Adsorption percentages were calculated by means of UV-VIS spectroscopy, considering the adsorption peak decrease respect to the bare solution at time 0. Wavelengths selected were 590 nm (CV), 555 nm (RH), and 485 nm (OII).

Some main points emerged from the adsorption results (Figure 8): in the case of both hydrogels and films, and for all the dyes measured, BBS-GC-containing gels always captured more contaminant than their corresponding control samples, due to the presence of available BBS-GC functionalities and to the more open and disordered network. Moreover, since better performances were reached in the presence of dyes bringing a positive charge, this suggested that the electrostatic interactions with negative carboxylate groups mainly drove the adsorptive process, whereas less significance can be attributed to the action of amino groups, which poorly enhanced the adsorption towards the negatively-charged OII. This evidence can be explained by the higher availability of free carboxylate groups (since they were provided by both BBS-GC and alginate) with respect to the amino groups (mainly brought by chitosan) which were essentially engaged in the formation of gel crosslinking. Furthermore, it was not possible to define a net advantage in using either hydrogels or dried films: despite their behaviour being similar in the presence of CV, the films performed better in the adsorption of RH, whereas in the case of OII solutions, the amount they were able to sequestrate was lower. Generally, the performances registered for the formulations examined seemed to be also related to the good water uptake capacities of chitosan-crosslinked gels, which encouraged the sequestration of the dyes’ solutions within the jelly networks.

## 3. Materials and Methods 

Sodium alginate (CAS 9005-38-3), partially N-deacetylated chitosan (DD = 75–85%) of medium molecular weight (CAS 9012-76-4), the dyes crystal violet (CV, >90%, CAS 548-62-9), rhodamine B (RH, >95%, CAS 81-88-9), orange II (OII, sodium salt, >85%, CAS 633-96-5), HCl (conc. 37%) and the spin-label 5-doxylstearic acid, free radical (5-DXS, CAS 29545-48-0) were purchased from Sigma Aldrich. The bio-based substances (BBS-GC) were isolated from composted urban biowastes, following a procedure reported in the literature [61]. 

Regarding sample synthesis, sodium alginate aqueous solution (2 wt.%) was put either in a glass open vial (28 × 57 mm) or a glass Petri dish (60 × 15 mm) and mixed with the BBS-GC solution (2 wt.% in water). Subsequently, the crosslinker was added (chitosan aqueous solution, 2 wt.% in 2 vol.% HCl) at a volume ratio of alginate: BBS-GC: chitosan = 2:1:2. The process of crosslinking took about 30 min. After rinsing with MilliQ™ water (see cleaning methods below), the gels obtained in the vials were kept hydrated, whereas those obtained in Petri dishes were put in a fume hood overnight at RT to allow the water to evaporate, thus obtaining dried films. Therefore, the differences between the hydrogels and dried films are the water content and the shape, being equal in the amount of active species. Gels without BBS-GC were also produced as reference samples, with a volume ratio of alginate:chitosan = 2:2. 

To wash the gels after the crosslinking, samples were soaked in 10 mL of MilliQ™ water and, then, gently shaken in rotating apparatus for ten minutes (10 rpm), repeating the procedure ten times per sample until the pH of the washing solutions passed from acid (due to the presence of chitosan solution excess) to neutral. The pH was monitored with a combined glass electrode connected to a Metrohm 713 pH-meter. A double-beam UV-visible spectrophotometer CARY 100 SCAN-VARIAN and a quartz cell with 1 cm path length were used to analyse the solutions derived from the repeated washing cycles, detect the loss of BBS-GC (following the absorption at 280 nm) and, thus, evaluate the stability of the samples containing BBS-GC.

Scanning electron microscopy (SEM) analysis was carried out on the dried films using a ZEISS EVO 50 XVP with LaB_6_ source, equipped with detectors for secondary electrons and energy dispersive X-ray spectrometry (EDS) probe for elemental analyses. Samples were covered with a gold layer of ca. 15 nm of thickness before the analysis to prevent charging effects (Bal-tec SCD050 sputter coater).

Fourier transform infrared (FTIR) spectra were recorded in transmission mode on a Bruker Vector 22 spectrophotometer equipped with a Globar source and deuterated triglycine sulphate (DTGS) detector. Spectra of dried films were collected in the 4000–400 cm^−1^ range, with 128 scans and resolution 4 cm^−1^, whereas the spectra of alginate, chitosan and BBS-GC powders were obtained after dispersing the samples in KBr (1:20 weight ratio).

Thermogravimetric analyses (TGA) and differential thermal analyses (DTA) were performed with a TA Instruments TGA Q600. The measurements were carried out in an open alumina pan, under nitrogen atmosphere and using a heating ramp of 10 °C min^−1^ from 40 to 800 °C. Measurements were performed on fragments of dried films (ca. 10 mg) and on the reference powders (i.e., neat alginate, chitosan and BBS-GC). Thermal profiles of hydrogels were also followed from 30 to 200 °C in order to assess the amount of water entrapped in the 3D networks. Prior to the analysis, gels were fast and gently compressed in laboratory paper to remove the excess of water.

The swelling properties were evaluated on dried films in terms of water uptake, by adding dropwise MilliQ™ water and measuring the weight increase (at 22 °C) until complete water saturation. The swelling ratios of the samples were calculated according to Equation (1):

S = W_1_/W_0_ (1)

where W_1_ indicates the mass at saturation and W_0_ corresponds to the initial dry mass. Furthermore, the time dependence of swelling ratio was determined maintaining the film samples in water at 22 °C. After pre-determined periods, the film was removed, gently compressed between filter papers (to remove the excess of water), then weighed and returned to the water bath [62].

Electron paramagnetic resonance (EPR) experiments were performed in an ESP300E Bruker X-band spectrometer. Hydrogels, with and without BBS-GC, were prepared as previously described but in the presence of 5-doxylstearic acid (10% *v*/*v*). After adding the cross-linker, the mixture was rapidly transferred into a capillary quartz tube, and the EPR spectrum was recorded using the following parameters: frequency = 9.78 GHz, microwave power = 5 mW, centre field = 3480 G, sweep width = 80 G, receiver gain = 1 × 10^5^, modulation amplitude = 0.5 G, conversion time = 40.96 ms. The mixture of 5-doxylstearic acid-alginate was used as control in the tests.

Rheological measurements to determine storage and loss moduli (G’ and G’’) were performed on a TA DISCOVERY RH1 instrument at 25 °C with a plate/plate configuration (steel upper geometry, 20 mm in diameter), keeping plates 500 µm apart. Samples were prepared ad hoc (disks of 9 mm diameter and 4 mm thickness) and analysed just after crosslinking. The lower plate was covered with abrasive paper to prevent the gel from gliding between surfaces. Analyses were performed in frequency sweep from 0.3 to 100 rad/s, as suggested in literature for hydrogels [63,64,65,66], with strain 1%, acquiring 5 points/decade. Before doing the measurements, preliminary tests were carried out in order to verify that the suggested parameters fit/correspond to the linear viscoelastic region of the samples.

The adsorption ability of both hydrogels and dried films was tested towards 5 mL of different dye solutions (10 mg L^−1^) at 22 °C. This way, the adsorbent/solution ratio was 2 g adsorbent/mg of dye, intended as the amount of precursor used for hydrogel/film preparation in relation to the weight of the selected dye in the starting solution. Selected dyes were orange II (OII), crystal violet (CV) and rhodamine B (RH). The kinetics of dye adsorption was followed for 1 h using a double-beam UV-visible spectrophotometer CARY 100 SCAN-VARIAN, measuring the absorption peak decrease at 485 nm for OII, 590 nm for CV and 555 nm for RH.

## 4. Conclusions

In this study, hydrogels and dried films were prepared using the physical interaction between alginate (polyanion) and chitosan (polycation). For the first time, to increase their adsorption ability, a lignin-derived water-soluble substance (BBS-GC) obtained from composted biowaste was included in the gel networks. Results from rheological tests evidenced that, for all the formulations, a stable 3D network was formed. Nevertheless, the inclusion of biowaste-derived macromolecules significantly influenced most of the physicochemical characteristics and the adsorbing features of BBS-GC-containing materials with respect to the controls (alginate-chitosan gels). BBS-GC complex structure caused a more open and disordered organization of the 3D (bio)polymeric main framework formed by alginate and chitosan chains. This rearranged network seemed to better allocate the differently charged dyes chosen as model pollutants in the adsorption experiments, namely orange II (negatively-charged), crystal violet (positively-charged) and rhodamine B (zwitterionic). The greatest effectiveness of BBS-GC-added samples towards molecules bringing a positive charge was ascribed to the electrostatic interactions with negative carboxylate groups, which BBS-GC are rich with. The use of chitosan as hygroscopic component favoured the adsorption performances due to the induced water uptake features, not affected by the hydrophobic moieties provided by biowaste-derived substances. In conclusion, in order to tailor the adsorbing features of gels for wastewater remediation, an additive can be contemporarily modulated in terms of steric effects, hydrophobic/hydrophilic character and availability of free and active functional groups. This approach results particularly convenient and sustainable when the additive derives from residual biomass.

## Figures and Tables

**Table 1:** Hydrogels water content (wt.%) evaluated using TGA and swelling ratio values found by water uptake experiments on dried films.

Sample Compositions (Hydrogels)	Water (%)	Sample Compositions (Dried Films)	Swelling Ratio
Alginate + Chitosan	98	Alginate + Chitosan	16
Alginate + Chitosan + BBS-GC	97	Alginate + Chitosan + BBS-GC	13

## Abbreviations

BBS-GC	Biowaste-derived substances isolated from green compost
CV	Crystal Violet
DTA	Differential Thermal Analysis (DTA)
EPR	Electron Paramagnetic Resonance
F-Alg/Chito	Film of Alginate and Chitosan
F-Alg/Chito/BBS	Film of Alginate, Chitosan and BBS-GC
FTIR	Fourier transform infrared spectroscopy
H-Alg/Chito	Hydrogels of Alginate and Chitosan
H-Alg/Chito/BBS	Hydrogels of Alginate, Chitosan and BBS-GC
OII	Orange II
RH	Rhodamine B
RT	Room Temperature
SEM	Scanning electron microscopy
TGA	Thermogravimetric analyses
